# Minimally Invasive Left Ventricular Assist Device Implantation: A Systematic Review of Current Evidence on Clinical Outcomes and Surgical Approaches

**DOI:** 10.3390/medsci13030173

**Published:** 2025-09-04

**Authors:** Baglan Turtabayev, Seitkhan Joshibayev, Umit Kervan, Samat Zharmenov, Yerbol Ustemirov, Almas Begdildayev, Gali Iskakbayev

**Affiliations:** 1 Department of Surgical Diseases, Kazakhstan’s Medical University “KSPH”, Almaty 050012, Kazakhstan; 2 Research and Clinical Center for Cardiac Surgery and Transplantology, Taraz 080000, Kazakhstan; 3 Ankara City Hospital Department of Cardiovascular Surgery, University of Health Sciences, Ankara 06560, Turkey

**Keywords:** left ventricular assist device, LVAD, minimally invasive surgery, thoracotomy, cardiac surgery, outcomes, survival rates

## Abstract

Background/Objectives: Minimally invasive cardiac surgical (MICS) approaches to the implantation of left ventricular assist devices (LVADs) have gained increasing interest as alternatives to full median sternotomy (FS), particularly in patients with prior cardiac surgeries or elevated surgical risk. However, evidence regarding their safety, feasibility, and clinical outcomes remains fragmented. This systematic review aimed to evaluate the effectiveness and safety of minimally invasive techniques for LVAD implantation in comparison to standard sternotomy, with a focus on mortality, perioperative complications, intensive care unit (ICU) stay, and infection rates. Methods: A comprehensive literature search was conducted in PubMed, Web of Science, Science Direct, Cochrane Library, and Google Scholar up to 1 January 2025. Studies were included if they reported on adult patients undergoing LVAD implantation via minimally invasive thoracotomy or sternotomy-sparing approaches, with or without comparator groups. Data were extracted and synthesized qualitatively; the Newcastle–Ottawa Scale (NOS) was applied to assess the methodological quality of the included cohort and retrospective comparative studies. Results: A total of 12 studies involving 1448 patients were included (584 received MICS and 862 received FS). MICS techniques have demonstrated comparable short and mid-term survival outcomes, with trends toward reduced ICU stay, fewer reoperations for bleeding, and lower incidence of driveline infections. Some studies reported longer operative and cardiopulmonary bypass times in the MICS group. Among high-risk cohorts, such as patients with prior sternotomies or significant comorbidities, MICS was associated with lower morbidity and acceptable safety profiles. However, heterogeneity in patient selection, surgical protocols, and outcome definitions limited quantitative synthesis. Conclusions: Minimally invasive LVAD implantation is a viable alternative to conventional sternotomy in selected patient populations. While current data suggest favorable perioperative outcomes and equivalent survival, high-quality prospective studies are needed to confirm long-term benefits and to guide patient selection. MICS approaches should be considered within multidisciplinary teams experienced in advanced heart failure surgery.

## 1. Introduction

Surgical technologies for implantation of circulatory assist devices, in particular left ventricular assist devices (LVAD), began to develop in the late 1990s against the backdrop of a rapid increase in the number of patients with terminal heart failure and limited access to donor organs [1,2]. Initially, LVAD implantation was performed exclusively through a median sternotomy (MS) [3], but with the advent of the third generation of centrifugal pumps and miniaturization of designs, it became possible to perform interventions with minimal trauma, which opened the way to less invasive surgery (minimally invasive cardiac surgery, MICS) [4,5].

Over the past decade, there has been a steady increase in interest in MICS strategies in the context of LVAD [6], driven by the desire to reduce surgical trauma, perioperative complications, the need for artificial circulation, and postoperative rehabilitation times [7,8,9]. The widespread implementation of this technique in practice has been accompanied by the emergence of a series of large cohort studies since 2013, as well as systematic reviews and meta-analyses [10,11,12,13], which compared MICS and traditional sternotomy access for a number of clinical and surgical outcomes.

Despite the growing body of evidence, a number of unresolved issues remain. Most studies report high population heterogeneity, do not always stratify correctly by severity of the condition (Interagency Registry for Mechanically Assisted Circulatory Support (INTERMACS) profiles) [14], and the impact of a less invasive approach on key parameters such as right ventricular failure, early mortality, transfusion requirements, and postoperative infections remains controversial [15]. In addition, published studies often lack a standardized approach to defining complications and using auxiliary techniques such as cardiopulmonary bypass or right ventricular support [16,17]. Finally, a significant proportion of studies are conducted on small samples, mainly in a single center, which limits the generalizability of the results [18,19,20].

Studies published in recent years allow us to re-evaluate the relevance of MICS in the context of modern clinical reality, with new intensive care standards and protocols without cardiopulmonary bypass [21,22]. In connection with the above limitations, this work is aimed at a systematic analysis of published data in order to objectively assess the safety and clinical effectiveness of a less invasive surgical approach compared to traditional sternotomy in LVAD implantation. Particular attention is paid to the analysis of mortality, the incidence of right ventricular failure, postoperative infections, blood loss, reoperations and the length of stay in the intensive care unit.

## 2. Materials and Methods

The study protocol was registered in the PROSPERO International Prospective Register of Systematic Reviews of the National Institute for Health Research (ID: CRD420251102537) [23].

### 2.1. Search Strategy

An initial search of the PROSPERO database for registered protocols of comparable systematic reviews and meta-analyses identified one relevant registration: the protocol and subsequent publication by Zhang et al. (2021) comparing outcomes after implantation of ventricular assist devices using minimally invasive and standard sternotomy approaches [24]. This meta-analysis included studies up to mid-2020, primarily on second-generation devices (HeartWare and HeartMate II, Framingham, MA, USA), with high heterogeneity in access type and operative technique. In contrast to the study by Zhang et al. [24], our review focuses on publications from recent years, reflecting the latest data on the use of third-generation devices as well, and evaluates a wide range of postoperative clinically important outcomes, including 30-day mortality, severe right ventricular failure, need for reoperations and transfusions, length of stay in the intensive care unit, and the incidence of infectious complications. Our analysis also emphasizes patients with repeat sternotomies and high surgical complexity.

**Figure 1:**
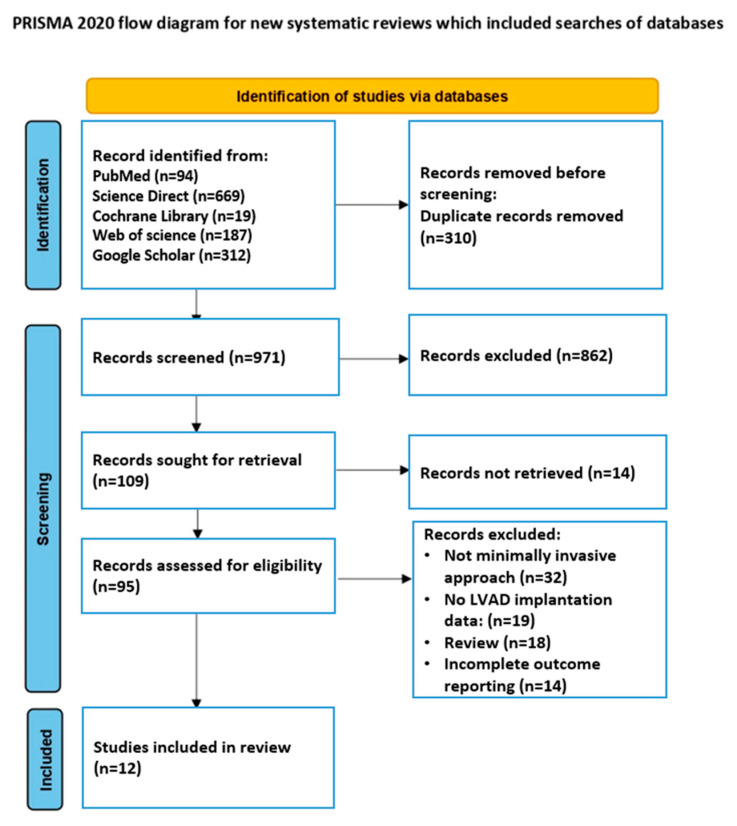
Flow chart of the PRISMA study selection process.

This systematic review was conducted and reported in accordance with the PRISMA 2020 (Preferred Reporting Items for Systematic Reviews and Meta-Analyses) guidelines [25]. A PRISMA 2020-compliant flow diagram (Figure 1) and the completed PRISMA checklist (Table S1) are available in the Supplementary Materials.

The search focused on studies evaluating postoperative clinical outcomes in patients who underwent left ventricular assist device (LVAD) implantation using various surgical approaches, including minimally invasive techniques. To ensure the relevance of the review to current surgical realities, the literature search was limited to publications from 2019 to 1 January 2025. This time range allows us to cover the most relevant studies reflecting the introduction of third-generation devices and the accumulation of comparative data on MICS approaches. The search was performed in five international bibliographic databases: PubMed, Science Direct, Cochrane Library, Web of Science and Google Scholar. A total of 1281 unique studies were identified. After removing duplicates, primary and full-text screening, taking into account the inclusion and exclusion criteria, 12 original studies were included in the final systematic analysis. In addition, a manual search of references in the bibliographies of relevant publications was performed. Gray literature (dissertations, preprints, posters, conference abstracts) was excluded. Only full-text peer-reviewed original studies in English were included in the review. Search commands included the following combinations of keywords and operators: “left ventricular assist device” AND “minimally invasive surgery”, “LVAD” AND “less invasive approach”, “LVAD implantation” AND “thoracotomy” AND NOT “sternotomy”, as well as relevant MeSH terms (“Heart-Assist Devices” [MeSH], “Thoracotomy” [MeSH], “Minimally Invasive Surgical Procedures” [MeSH]), taking into account differences in terminology between databases. The selection of search strategies was adapted to the interface of each platform.

### 2.2. Eligibility Criteria

The systematic review included original studies that met the following criteria: type—retrospective or prospective cohort studies, as well as comparative studies and RCTs (if available); population—adult patients with end-stage heart failure who received not only first and second-generation LVADs, but also third-generation LVADs (HeartMate 3); intervention—minimally invasive surgical approach; comparison—traditional median sternotomy; outcomes—at least one clinically important postoperative indicator (mortality, severe right ventricular failure, need for right ventricular assist device (RVAD), reoperation for bleeding, transfusion volume, ICU duration, infections, length of hospital stay). Articles that did not contain stratified data by type of surgical approach, publications without peer review, studies with duplicate data, non-English language sources, and studies that included minors were excluded (Table 1).

### 2.3. Selection of Studies and Data Extraction

After removing duplicates, two independent reviewers performed a stepwise screening of identified records based on titles and abstracts. Publications that met the inclusion criteria at this stage were selected for full-text analysis. The final decision on inclusion was made after an in-depth assessment of the article content against pre-defined eligibility criteria.

Data extraction was also performed by two independent investigators. In case of discrepancies in interpretation or coding of information, a third expert was involved to reach a unified conclusion. For each included study, the following characteristics were recorded: first author’s name, year of publication, country/region of conduct, study design, sample size in comparison groups, type of surgery (minimally invasive or traditional), presence of postoperative outcomes (including mortality, incidence of right ventricular failure, use of RVAD, volume of blood loss/transfusion, length of ICU and hospital stay, infectious complications), as well as data on the duration of follow-up and the incidence of heart transplantation, if available. Where necessary, missing data were clarified manually from the original text or tables. All extracted information was verified, structured and converted to a standardized format for subsequent analysis.

### 2.4. Risk of Bias (Quality) Assessment

To analyze the internal reliability of the included studies, the Newcastle–Ottawa Scale (NOS) in its original modification for cohort and retrospective comparative studies was used, which corresponds to the recommendations of the Cochrane Collaboration Working Group on the Appraisal of Non-Randomized Trials [26]. This scale allows for a quantitative assessment of the risk of bias in three key domains: selection of participants (maximum 4 points), comparability of study groups (maximum 2 points), and outcome assessment (maximum 3 points), with a total range from 0 to 9 points. A high total score reflects better methodological validity and a lower risk of systematic error.

The assessment procedure was performed by two reviewers independently. Any discrepancies were resolved by discussion until a full consensus was reached; a third expert was involved if necessary. In accordance with the adopted thresholds, studies with a score of ≥7 were classified as high-quality, 5–6 as moderate quality, and ≤4 as low qualities. The results of the quality assessment were documented in a separate table and taken into account when interpreting the collected data. Studies with a high risk of bias were not excluded from the review, but in each case their contribution to the overall conclusions was critically analyzed, taking into account the context of the design and the presence of confounders (Table 2).

Given the significant heterogeneity in the included study designs, clinical populations, and outcome measures, meta-analysis and pooled quantitative estimates were not performed. Formal procedures for assessing sensitivity, risk of publication bias, and certainty of evidence were not performed. Instead, heterogeneity was analyzed qualitatively by comparing clinical outcomes according to surgical approach (minimally invasive versus median sternotomy), sample design, devices used, rates of concomitant interventions, and differences in management protocols. The results should be interpreted with caution given the synthetic nature of the review and the potential for residual methodological bias.

## 3. Results

### 3.1. Included Study Characteristics

The final systematic review included 12 original studies that met the established selection criteria. All publications had a retrospective or prospective cohort design and were published between 2019 and 2025. The geography covered European countries (Poland, Germany, Turkey, Hungary), North America (USA, Canada), as well as multicenter international studies, including one five-center and one twenty-three-center study.

The total number of patients who underwent minimally invasive LVAD implantation was 584, while 862 patients were analyzed in the comparison groups operated using full median sternotomy. The sample size in individual studies ranged from 11 to 204 patients per group. All studies used third-generation devices, primarily the HeartMate 3, and some used the HVAD.

The primary clinical outcomes assessed in most studies (n = 10) included postoperative mortality (30-day, 6-month, 1 and 2-year), incidence of right ventricular failure and RVAD use, intra and postoperative transfusion volumes, length of intensive care unit stay, incidence of infectious complications (including wound and drainage line infections), as well as surgical time and need for reoperation for bleeding. Two studies additionally assessed quality of life and functional status of patients after implantation.

It should be noted that the review included two independent studies, the first author of which is Gosev (2019; 2024) [34,38]. Despite the coincidence of the lead author, these studies differ significantly in design (retrospective single-center and prospective multicenter), sample, methodology, and time frame. Both studies were assessed as methodologically distinct and therefore were included in the review as independent sources of data. Detailed characteristics of the included studies are presented in Table 3.

#### Baseline Patient Characteristics Across Studies

Table 4 presents the demographic and clinical characteristics of patients from the 12 studies included in the analysis. Across studies, mean patient age ranged from 44 to 64 years, with 72–95% males. Prior cardiac surgery prevalence varied from 12% to 73%, showing statistically significant differences only in some studies. INTERMACS profiles and comorbidities—including ischemic or dilated cardiomyopathy, chronic renal failure, COPD, diabetes, and hypertension—displayed variability but no systematic differences between FS and MICS in most cohorts. Some studies reported higher preoperative ECMO or IABP use in FS groups [27,28,29,30,31,32,33,34,35,36,37,38].

### 3.2. Clinical Outcomes and Comparison of Minimally Invasive Approach and Sternotomy

The 12 included studies analyzed postoperative outcomes in patients undergoing LVAD implantation via a minimally invasive approach (MICS) compared with full median sternotomy (FS). Ten studies included a control group, allowing a direct comparison between the two surgical methods.

Mortality: Thirty-day mortality in the MICS groups ranged from 0% to 13.6% and was generally lower or comparable to FS. In the study by Özer [31], early mortality was 0% in the MICS group and 13.3% in the FS group. Sun [37] reported 3.8% versus 14.9% (*p* = 0.02). Long-term survival was similar in both groups. In the multicenter HM3 SWIFT study [38], six-month survival was 89% for MICS and 90% for FS, with no difference in event-free survival (85.0% vs. 86.2%; hazard ratio 1.01; 95% CI 0.58–2.10). Jawad et al. [27] reported 1-year cumulative mortality of 22.5% (95% CI 12.8–33.8) and 2-year cumulative mortality of 25.2% (95% CI 14.5–37.4) after MICS, with no significant difference compared to FS (subdistribution hazard 0.904; *p* = 0.77). Antończyk et al. [33] showed mean survival of 633.2 ± 426.8 days in MICS versus 430.0 ± 291.8 days in FS, without statistical significance.

Blood loss and reoperations: MICS reduced intra and postoperative bleeding in several studies. Reoperations due to bleeding were less frequent in MICS in Jawad [27] and Gosev [34]. Özer [31] reported a high reoperation rate of 23.3%, but no early deaths occurred. The HM3 SWIFT study [38] found similar rates in both groups (10.8% vs. 11.8%).

ICU length of stay: Most studies showed shorter ICU stay for MICS patients. Jawad [27] and Ayers [36] reported reduced ICU days. In Sun [37] and Gosev [38], the difference was not significant, especially in centers with high surgical volume and established MICS protocols.

Infectious complications: Infection rates were generally lower in MICS [27,28,33,34]. Antonczyk [33] observed no wound infections in MICS (0% vs. 6%). Kervan [30] reported a higher infection rate of 44% in MICS, mainly at the thoracotomy site (27%), but systemic infections were not increased in any study.

Right ventricular failure (RVF) and RVAD use: Most studies showed lower RVF and RVAD use in MICS. Saeed [36] reported RVF 18.6% vs. 29.9% and RVAD use 8.2% vs. 18.6%. Gosev [34] observed RVF 7% vs. 28%. However, a later Gosev study [38] found higher RVAD use in MICS (13.7% vs. 5.4%, *p* = 0.02) due to changes in patient selection and clinical approach.

Functional status and surgery duration: Few studies assessed functional outcomes. Gosev [38] reported improvements in New York Heart Association (NYHA) class and 6-min walk test in both groups. Surgery time was longer in MICS; for example, HM3 SWIFT reported 396 min versus 284 min for FS, reflecting technical complexity and the learning curve.

An aggregated analysis of key outcomes is shown in Table 5, covering seven major parameters: 30-day mortality, six-month survival, reoperation for bleeding, ICU stay, infectious complications, RVF, and RVAD use.

High-risk patient subgroup: Reichart et al. [28] and Antonczyk et al. [33] studied MICS in high-risk patients. Antonczyk [33] included 54% of patients with prior cardiac surgery (Table 6). Peripheral cannulation was frequent (femoral artery 92%, vein 100%) and surgery was longer (median 367.5 min). Reichart [28] used MICS in patients with preserved lung function and no need for other interventions; 72.7% had peripheral cannulation and none required conversion to FS. Both studies confirmed MICS is feasible and safe in challenging cases.

## 4. Discussion

This systematic review summarizes the clinical outcomes of a minimally invasive approach (MICS) for left ventricular assist device (LVAD) implantation, including the HeartMate 3 and HVAD. With increasing interest in minimizing surgical trauma, MICS is increasingly being considered as an alternative to traditional full sternotomy. Our analysis confirms that MICS is characterized by high clinical effectiveness, comparable or superior to standard approaches in several key outcomes.

Comparison of MICS and sternotomy on major clinical outcomes

The general data demonstrate that the use of minimally invasive access does not increase, and in some cases decreases, postoperative mortality, both short-term (30 days) and over 1–2 years of follow-up [27,31,33,37,38]. This confirms the promise of this approach even in the group of patients with increased surgical risk, including repeated interventions [33] and severe comorbidities [28,30]. In particular, patients undergoing repeat sternotomy, those with frailty or obesity, and patients receiving LVAD as a bridge to transplantation appear to benefit most from MICS. Minimizing surgical trauma in these subgroups reduces postoperative complications, facilitates recovery, and preserves anatomical structures critical for future interventions [33,34]. Furthermore, one study [29] showed that in patients with end-stage Congestive Heart Failure (CHF) receiving LVAD as a bridge to transplant, the minimally invasive technique was associated with a lower incidence of donor-specific antibodies (4% vs. 36%, *p* = 0.006) and lower transfusion requirements at the time of transplant (4 [IQR 2–7] vs. 7 [IQR 4–8] units, *p* = 0.045), with comparable in-hospital mortality. These data highlight the potential of MICS to improve long-term outcomes in high-risk patients. Numerous studies have shown a significant reduction in the volume of intraoperative blood loss, the frequency of transfusions and reoperations due to bleeding in the MICS group [27,31,32,34,35]. A significant reduction in the length of stay in the intensive care unit and in the hospital was also noted [27,31,38]. These reductions have important implications for resource utilization and planning accelerated postoperative recovery (Enhanced Recovery After Surgery (ERAS) protocols). Shorter ICU and hospital stays not only decrease costs but also allow patients to resume social and physical activities sooner, which may improve long-term functional outcomes [39,40,41].

Infectious complications and wound healing

The incidence of infectious complications, especially those related to the drainage line and the postoperative wound, was significantly lower with MICS [27,28,33,34]. This is likely due to the smaller surgical access area, reduced risk of contact with the prosthesis, and limited exposure to sterile areas. Previously published data also suggest an advantage of MICS in reducing the incidence of mediastinitis and osteomyelitis of the sternal bone [42,43,44]. While most studies demonstrated a reduction in infectious complications with MICS, one single-center study by Kervan [30] reported a markedly higher infection rate, particularly involving the thoracotomy site. This may be attributable to specific local wound care protocols, surgical technique, or patient-related factors such as comorbidity burden. Notably, driveline exit site infections—regardless of surgical approach—remain a challenging complication to prevent, as the driveline traverses the skin in the same manner in both MICS and full sternotomy [45].

Right ventricular failure and RVAD

Particular attention should be paid to the discussion of right ventricular failure (RVF) and the need for a right ventricular assist device (RVAD). Despite the heterogeneous data, some studies indicate a higher frequency of RVAD in the MICS group [38], which may be associated with a more severe baseline status of patients selected for MICS. At the same time, other studies, on the contrary, record a tendency towards a lesser severity of RVF, explaining this by the anatomical preservation of the pericardium and minimal dissection with a minimally invasive technique [33,37]. Systematic reviews of recent years also note that incomplete pericardiotomy in MICS allows for a decrease in the mechanical load on the right ventricle and improves its adaptation after LVAD inclusion [46,47,48,49,50].

Complications associated with bleeding and blood transfusion

Despite longer operative time and CPB in MICS in some publications [31,33,38], this approach did not increase the frequency of blood transfusions or the volume of blood loss. Some data, on the contrary, indicate a lower need for red blood cells and fresh frozen plasma [34,35], which is of key importance in patients with anemia or impaired hemostasis [51,52,53,54,55,56]. Data from Riebandt et al. [29] show that MICS LVAD—in particular implantation via LIS—is accompanied by the development of donor-specific antibodies in the sternotomy area, as well as low transfusion measures during transplantation, while maintaining progressive in-hospital mortality. Traumatic access may reduce sensitization and facilitate subsequent operations, especially in the setting of a limited donor pool.

Impact of surgical experience

A number of authors note that the surgeon’s experience and the annual volume of operations at the center directly affect the results of MICS. As shown by Antończyk et al. [33], the operating time for minimally invasive LVAD implantation in centers at the stage of mastering the technique was significantly higher (median 367.5 min versus 265 min for sternotomy; *p* < 0.001). A similar trend is confirmed in the multicenter HM3 SWIFT study (Gosev et al. [38]), where the length of hospitalization in the thoracotomy group was slightly longer (median 20 days versus 17 days; *p* = 0.03), which the authors associate with the learning curve and adaptation of teams to the new technique. At the same time, in high-volume centers with established protocols, operative times and perioperative outcomes approach those of sternotomy, and complication rates—including right ventricular failure and bleeding—are comparable or lower. For example, Jawad et al. [27] reported in a multicenter study (n = 405) that centers with >20 MICS-LVADs per year had shorter operative times (mean reduction of 38 min) and lower rates of conversion to sternotomy compared with low-volume centers. Saeed et al. [36] indicate that the learning curve for MICS is approximately 15–20 procedures, after which complication rates decrease and hemodynamic outcomes improve.

Functional and subjective indicators

Several studies have additionally assessed the recovery of functional status and quality of life. In particular, improvement in the New York Heart Association (NYHA) scale, an increase in the distance in the 6-min walk test and an increase in scores in questionnaires Kansas City Cardiomyopathy Questionnaire (KCCQ, EQ-5D) were observed in both groups, without significant differences [38]. In the work of Ozer et al. [31], after 12 months of follow-up, functional recovery (six-minute walk test) was maintained and quality of life according to the KCCQ questionnaire stabilized. Despite the lack of large-scale RCTs on this issue, the accumulated data confirm that the effect of MICS on long-term outcomes requires further multicenter study. Improvements in NYHA functional class, six-minute walk distance, and patient-reported quality of life suggest that MICS preserves or enhances postoperative function compared with full sternotomy [31,37]. These benefits are particularly relevant in high-risk patients or those awaiting heart transplantation, where less invasive access may reduce sensitization, facilitate future surgery, and optimize long-term outcomes [29,57,58].

Applicability of MICS in complex clinical scenarios

Of particular note are studies in which MICS was used in patients with high surgical risk, including those with COPD, obesity, fibrosis from previous surgeries, or in conditions of limited access to sternotomy [33,34]. In such clinical situations, minimizing chest trauma may be a determining factor in a successful outcome [39,59,60]. It is important to note that some authors suggest using MICS as a preferred approach in patients awaiting heart transplantation to facilitate subsequent surgery [59,60,61,62].

Methodological limitations and potential impact on conclusions

The included studies differed in design, setting, and patient selection. Some were retrospective, others prospective; some were single-center, others multi-center. Patients varied in baseline risk, and surgical experience and hospital volume were not consistent. Inclusion criteria, follow-up length, device types, surgical protocols, and perioperative care also differed. This makes direct comparison difficult and means the results should be seen as indicating likely trends rather than definitive proof. Despite this, pooled data from Table 5 show clear patterns: 30-day mortality was lower with MICS (4.8%, range 0–13.6%) than FS (12.1%, range 10.3–14.9%), ICU stay was shorter (6.3 days, range 3.7–13.5 vs. 8.4 days, range 4–13.5), and reoperations for bleeding were less frequent (8.0%, range 0–23.3% vs. 12.6%, range 5–23.3%). These trends appear across different centers and patient populations, supporting the potential benefits of MICS and highlighting the need for prospective trials.

### Limitations

This systematic review has a number of limitations that should be taken into account when interpreting the results. First of all, the included studies varied significantly in design, geography, population characteristics, and data collection methods. The sample included both single-center and multicenter observations, mostly retrospective in nature, with varying duration of follow-up and varying degrees of reporting completeness. Such methodological and clinical heterogeneity, including differences in the types of devices used (HeartMate 3, HVAD), prevented formal meta-analysis and limits the interpretation of aggregated indicators. In addition, it should be noted that none of the included studies was a randomized controlled trial. The use of propensity score matching methods, as in individual prospective studies, only partially compensates for selection bias, without completely eliminating the influence of unaccounted factors.

A separate problem is the limited data on functional and subjective outcomes. Only a few authors have assessed quality of life, level of physical activity or recovery of work capacity after discharge. At the same time, such parameters are of key importance for justifying the appropriateness of the choice of surgical approach in patients with a long predicted life expectancy with mechanical support. Some studies did not report complete baseline demographic and clinical data, which limits the ability to perform comprehensive risk-adjusted comparisons between surgical approaches.

It is also worth emphasizing that stratification by the level of surgical risk was not performed in all studies, which creates difficulties in interpreting data in patients with repeated interventions, severe comorbidities, or unstable hemodynamic status. The lack of unified inclusion and exclusion criteria increases the likelihood of bias and limits the reproducibility of the results obtained in other centers.

An additional limitation is the poor presentation of long-term outcomes. In most cases, follow-up was limited to 6 months, and only a few studies reported 2-year survival rates. This significantly limits the understanding of long-term risks such as infectious complications, thrombotic events, or progression of right ventricular failure. Prospective studies with long-term follow-up of quality of life and functional rehabilitation are also required.

## 5. Conclusions

The results of a systematic review demonstrate the high clinical viability of minimally invasive implantation of left ventricular assist devices (LVADs) as an alternative surgical approach in certain patient categories. With comparable mortality rates, infectious complications, and the need for repeated surgical interventions, this technique has demonstrated advantages in a number of studies, including a shorter stay in the intensive care unit, a decrease in the volume of intra- and postoperative blood transfusions, and a decrease in the incidence of surgical wound infections.

Minimally invasive access is especially indicative in patients with high surgical risk, including those previously operated on, with severe concomitant pathology, and in conditions of limited anatomical possibilities. However, the heterogeneity of study designs, limited duration of observation, and heterogeneity of the methodological approaches used do not allow for unambiguous generalizations. Therefore, the introduction of minimally invasive LVAD implantation into widespread clinical practice should be accompanied by strict patient selection, standardization of surgical protocols, and a system of professional training for surgeons.

For final validation of the identified advantages, prospective randomized studies with a long-term follow-up period, risk stratification, and assessment of functional outcomes and quality of life are needed. Such an approach will help determine the place of minimally invasive technology in the modern strategy of treating terminal heart failure and formulate well-founded clinical recommendations for various groups of patients.

## Figures and Tables

**Table 1:** Inclusion and exclusion criteria for publication selection.

Category	Inclusion Criteria	Exclusion Criteria
Type of research	Original articles: retrospective and prospective cohort studies, comparative observational studies, RCTs (if available)	Reviews, meta-analyses, case reports, letters, posters, dissertations, conference proceedings, untraceable sources (gray literature)
Patients	Adult patients (≥18 years) with end-stage chronic heart failure who have undergone LVAD implantation	Studies Involving Children/Adolescents (<18 Years)
Intervention	Minimally invasive approaches: mini-thoracotomy, mini-sternotomy, bilateral thoracotomy, etc., without the use of full median sternotomy	Lack of data on the type of access, impossibility of stratifying the results by the type of intervention
Outcomes	Presence of at least one postoperative outcome: 30-day mortality, RVAD, bleeding, transfusion, ICU, infection, length of hospital stay	Articles without indication of clinical outcomes or aggregated data without separation by access groups
Language and access	Publications in English with access to full text	Non-English articles without translation; full text unavailable
Originality of data	Primary source with clinical results	Duplicate publications without new data

**Table 2:** Qualitative Assessment of Included Studies Using the Newcastle–Ottawa Scale (NOS).

Study (Author, Year)	Selection (Max 4)	Comparability (Max 2)	Outcome (Max 3)	Total Score (Max 9)
Jawad et al. (2021) [27]	4	2	3	9
Reichart et al. (2019) [28]	4	1	2	7
Riebandt et al. (2021) [29]	4	1	2	7
Kervan et al. (2021) [30]	4	1	2	7
Ozer et al. (2020) [31]	4	1	2	7
Pasrija et al. (2020) [32]	3	2	3	8
Antonczyk et al. (2024) [33]	4	1	2	7
Gosev et al. (2019) [34]	4	2	3	9
Ayers et al. (2020) [35]	4	1	2	7
Saeed et al. (2021) [36]	4	2	3	9
Sun et al. (2024) [37]	4	2	3	9
Gosev et al. (2024) [38]	4	2	3	9

**Table 3:** Outcomes of minimally invasive (MICS) vs. Conventional Approaches for LVAD Implantation.

No.	Authors (Year)	Country	Study Design	Sample Size (MICS/Comparator)	Mortality	Blood Loss/Reoperation	ICU Stay	Infection Rate	Key Findings
1	Jawad et al. (2021) [27]	Germany, Canada	Multicenter, retrospective, PSM	73/73	1-yr: 77.5% (MICS) vs. 77.3% (FS); 2-yr: 74.8% vs. 70.9%	Reoperation: 4.1% (MICS) vs. 6.8% (FS)	Median 4 (IQR 2–9.25) vs. 6 (IQR 3–13)	Driveline: 2.7% vs. 5.5%	MICS led to fewer reoperations, shorter ICU and hospital stay, and comparable long-term survival.
2	Reichart et al. (2019) [28]	Germany	Single-center, retrospective	22/53	30-day: 13.6% (MICS), 12.5% (STX); 2-yr: no difference	RBC transfusion (7d): 6.6 ± 6.3 (MICS) vs. 8.4 ± 9.5 (STX)	13.5 ± 25.2 days (both)	LVAD-related: 18.2% (MICS) vs. 41.7% (STX); Wound: 0% vs. 10.4%	MICS reduced infection and wound complications; survival comparable to sternotomy.
3	Riebandt et al. (2021) [29]	Austria	Retrospective cohort	27 (LIS)/19 (FS)	LIS: 7% vs. FS: 5% (*p* = 1.000)	4 (IQR 2–7) vs. 7 (IQR 4–8) units (*p* = 0.045); reoperation data not provided	Not reported	Not reported	LIS associated with reduced blood product use and significantly lower de novo DSA formation post-HTX (4% vs. 36%; *p* = 0.006). Survival and adverse events were similar.
4	Kervan et al. (2021) [30]	Turkey	Single-center, retrospective	41 (no comparator; early deaths excluded)	30-day: excluded	Blood loss: 582 ± 221 mL; 0% reop	6.4 ± 3.5 days	44% total (27% driveline, 17% incision site)	Lower bleeding and ICU stay with MICS; higher thoracotomy site infection.
5	Ozer et al. (2020) [31]	Turkey	Retrospective comparative (matched)	30/30	30-day: 0% (MICS) vs. 13.3% (FS)	Blood loss: 860 ± 458 mL (MICS) vs. 1210 ± 560 mL (FS); Reop: 23.3% (both groups)	3.7 ± 3.5 days (MICS) vs. 5.4 ± 4.6 days (FS)	Not reported	MICS had lower bleeding, shorter ICU stay, and no early mortality.
6	Pasrija et al. (2020) [32]	USA	Single-center, retrospective	11/12 (high-risk patients)	1st yr: 91% (MICS) vs. 42% (FS)	RBC units: 3 (IQR 2–7) vs. 6 (IQR 3–12)	Median 7 (IQR 5–20) vs. 12 (IQR 7–28)	Not reported	Less RVAD use, shorter CPB, and improved survival in MICS group (91% vs. 42%, *p* = 0.033).
7	Antonczyk et al. (2024) [33]	Poland	Single-center, retrospective	26/22	1-yr: 84% both; 2-yr: 84% (MICS) vs. 73% (STX), *p* = 0.79	More intraop FFP/platelets in MICS; reop bleeding: NS	Not reported	Wound: 0% (MICS) vs. 6% (STX); other: NS	MICS had longer OR time, fewer infections, similar survival; preferable in redo cases.
8	Gosev et al. (2019) [34]	USA	Single-center, retrospective	41/36	6-month: 93% vs. 77% (*p* = 0.088)	Reop: 5% vs. 20%; drain output: 1045 vs. 1403 mL	Median 4 (IQR 3–7)	SSI: 10%, Driveline: 2%	Lower RV failure (7% vs. 28%), transfusions, and faster recovery with MICS.
9	Ayers et al. (2020) [35]	USA	Single-center, retrospective	15/14	30-day: 6.7% (MICS) vs. 14.3% (FS)	RBC, plasma, platelets significantly less in MICS (*p* < 0.05)	Median 4 (vs. 13 in FS)	Not detailed	Lower RVF (*p* = 0.006), shorter LOS, fewer transfusions; no conversions to sternotomy.
10	Saeed et al. (2021) [36]	5 countries	Multicenter, retrospective	122/305	1st year: No difference	RHF: 18.6% vs. 29.9%, RVAD: 8.2% vs. 18.6% (*p* < 0.01)	Not reported	Not reported	LIS associated with less RHF and RVAD; survival similar.
11	Sun et al. (2024) [37]	Canada	Single-center, retrospective	78/94	30-day: 3.8% vs. 14.9% (*p* = 0.02)	No diff in severe RVF	Same: 8 days	Not reported	Less platelet transfusion, lower 30-day mortality; CPB avoidance may reduce complications.
12	Gosev et al. (2024) [38]	USA, Multicenter	Prospective multicenter, propensity-matched cohort	102 (MICS)/204 (sternotomy)	6-month: 11% (MICS) vs. 10.3% (sternotomy); non inferior (*p* < 0.0025)	Reoperation: 10.8% (MICS) vs. 11.8% (FS); CPB: 123 ± 65 vs. 84 ± 40 min	Median: 7 days (MICS) vs. 8 days (sternotomy); N.S.	Major infection: 38.2% (both groups); driveline infection: 8.8% (MICS) vs. 8.3%	Thoracotomy-based implantation was noninferior to sternotomy in survival, stroke, and major complications; longer OR time but similar ICU stay and adverse event rates. RVAD use higher in MICS group (13.7% vs. 5.4%; *p* = 0.02).

Common abbreviations used in the table include: MICS (minimally invasive cardiac surgery), FS (full sternotomy), LIS (less invasive surgery), STX (sternotomy), LVAD (left ventricular assist device), RVF (right ventricular failure), RVAD (right ventricular assist device), RHF (right heart failure), CPB (cardiopulmonary bypass), ICU (intensive care unit), OR (operating room), LOS (length of stay), SSI (surgical site infection), RBC (red blood cells), FFP (fresh frozen plasma), DSA (donor-specific antibodies), HTX (heart transplantation), CMP (cardiomyopathy), NS (not significant), and PSM (propensity score matching).

**Table 4:** Comparative characteristics of patients who underwent LVAD implantation through full sternotomy (FS) and minimally invasive access (MICS).

No.	Study	Age, Years (Mean ± SD) (FS/MIC S)	Males, % (FS/MIC S)	Previous Cardiac Surgeries, % (FS/MIC S)	INTERMACS (Mean ± SD or % I–III)	Key Outcomes/Comorbidity
1	Riebandt et al. [29]	58.3 ± 9.0/56.0 ± 7.9	84/82	Not reported	I–III: 68/70 (*p* = 0.887)	“Patients were comparable in preoperative characteristics, including kidney and liver function, INR levels (full sternotomy [FS]: 2.2 ± 0.9 vs. less invasive surgery [LIS]: 2.5 ± 0.5; *p* = 0.357), and assessment by the Model for End-Stage Liver Disease excluding INR (MELD-XI) (FS: 8.48 ± 4.22 vs. LIS: 10.35 ± 4.53; *p* = 0.159).”
2	Gosev et al., (SWIFT) [38]	58.4 ± 12.4/59.1 ± 13.4	83.8/81.4	28.4/27.5	32.4/31.4	All patients with advanced heart failure were excluded from the study if biventricular circulatory support or concomitant procedures were planned at the time of left ventricular assist device (LVAD) implantation, or if irreversible end-organ dysfunction or active infection was present.
3	Jawad et al. [27]	59 ± 11 (total cohort)	90 (total cohort)	23/14 (*p* = 0.08)	2.35 ± 1.13/2.83 ± 1.0 (*p* < 0.001)	ECMO before implantation: 19.6% total; long-term ECMO CS 9 ± 6 vs. LIS 5 ± 3 days (*p* = 0.02); Tricuspid valve insufficiency grade II-III27 FS (42.9%, n = 63)/MICS 26 (37.7%, n = 69) *p* = 0.106 Etiology of ischemic cardiomyopathy FS 37 (51.4%, n = 72)/MICS 40 (54.8%, n = 73) *p* =0.068
4	Reichart et al. [28]	52 ± 15 vs. 59 ± 11	95.4/83.3	Not reported	31.8%/68.8%, *p* = 0.01	Dilated CMP MICS 12 (54.5) STX 21 (43.7), Ischemic CMP MICS 6 (27.3) STX 20 (41.7)
5	Kervan et al. [30]	45.7 ± 13.7/44.8 ± 15.6	90/73	14/17 *p* = 0.03	INTERMACS 1 9/7% INTERMACS 2 24/20 *p* = 0.6 INTERMACS 3 25/24 *p* = 0.1	Dilated cardiomyopathy CS 53% MICS 68%, Ischemic cardiomyopathy CS 47% MICS 32%, Diabetes mellitus CS 31%, MICS 17% Chronic obstructive pulmonary disease CS 10%, MICS 7%
6	Ozer et al. [31]	49.1 ± 10.4/46.6 ± 11	93.3/83.3	13.3/30 *p* = 0.2	43.3/63.3	Hypertension MICS 12 (%40) FS 8 (%26.7) Cardiac indices, pulmonary vascular resistances, transpulmonary pressure gradients, central venous pressures, and pulmonary artery pressures were not statistically different between the two groups (*p* > 0.05)
7	Antończyk et al. [33]	53.7 ± 11.9/55.8 ± 10.7	No data	12/54 *p* < 0.001	Not reported	Among patients, ischemic cardiomyopathy was present in 45% of those undergoing full sternotomy (FS) and 77% in the minimally invasive cardiac surgery (MICS) group. Dilated cardiomyopathy was present in 36% of FS patients and 8% of MICS patients. Extracorporeal membrane oxygenation (ECMO) as a bridge to left ventricular assist device (LVAD) implantation was used more frequently in the FS group (11% vs. 0%, *p* = 0.03).
8	Ayers et al. [35]	63.6 ± 11.3/60.4 ± 9.2	79/93	50/73 *p* = 0.264	1.9 ± 0.9/2.4 ± 0.9 *p* = 0.120	Chronic renal insufficiency FS 6 (40%) MICS 5 (36%), Diabetes FS 5 (33%), MICS 7 (50%), COPD FS 3 (20%), MICS 3 (21%)
9	Saeed et al. [36]	58 ± 11 vs. 58 ± 12	There were no differences in mean age, gender, body mass index, diabetes mellitus, or history of hypertension between patients in the MICS group and the FS group. Preoperative use of ECMO and IABP was more common in the FS group, while off-pump implantation was more frequent in the MICS group. Other preimplantation variables, including creatinine levels, hemodynamic parameters, and tricuspid regurgitation, did not differ between the two groups.			
10	Sun et al. [37]	56.0 (47.0, 63.0)/54.0 (46.2, 62.0)	72.3/64.1	21.3/26.9 *p* = 0.39	1. 44 (46.8)/17 (22.1) 2. 26 (27.7)/29 (37.7) 3. 24 (25.5)/31 (40.3)	Ischemic cardiomyopathy (%): FS—41.5%, MICS—35.9%, *p* = 0.45, Dialysis dependence (%): FS—9.6%, MICS—10.3%, *p* = 0.88, Peripheral vascular disease (%):FS—2.1%, MICS—5.1%, *p* = 0.41, Insulin-dependent diabetes (%):FS—7.4%, MICS—9.0%, *p* = 0.93, Myocardial infarction ≤ 90 days (%):FS—25.5%, MICS—12.8%, *p* = 0.06, Congenital heart disease (%):FS—5.3%, MICS—11.5%, *p* = 0.14, Chronic disease mild (%):FS—10.6%, MICS—5.1%, *p* = 0.26
11	Pasrija et al. [32]	55 years (33–57)/55 years (36–64)	67/91	No data	75%—preoperative VAD or VA-ECMO, the rest INTERMACS 1 with inotropes and IABP/37%—preoperative VAD or VA-ECMO, the rest INTERMACS 1 (86% of them with IABP)	FS/MICS Ischemic etiology: 42%/27% Atrial fibrillation: 42%/46% Heart thrombus: 8%/9% Oncology: 0%/18% History of stroke: 8%/9% Diabetes mellitus: 67%/18% Obesity: 33%/46%
12	Gosev et al. [34]	57 years (43–64)/61 years (56.5–68)	76/78	24%/22% *p* = 0.084	Profile 1–41%/34% Profile 2–15%/8% Profile 3–39%/48% Profile 4–5%/9%	FS/MICS Ischemic etiology: 44%/47% Stroke or TIA: 7%/5% Chronic renal failure: 27%/39% Diabetes mellitus: 29%/38% COPD: 12%/14%

Common abbreviations used in the table include: FS—full sternotomy, MICS—minimally invasive cardiac surgery, BTT—bridge to transplantation, CS—conventional sternotomy, LIS—less invasive surgery, SS—sternal-sparing, TS—traditional sternotomy, INTERMACS—Interagency Registry for Mechanically Assisted Circulatory Support, NYHA—New York Heart Association functional classification, ECMO—extracorporeal membrane oxygenation, VA-ECMO—veno-arterial extracorporeal membrane oxygenation, IABP—intra-aortic balloon pump, VAD—ventricular assist device, LVAD—left ventricular assist device, CMP—cardiomyopathy, MELD-XI—Model for End-Stage Liver Disease excluding INR, TIA—transient ischemic attack, COPD—chronic obstructive pulmonary disease, BMI—body mass index, SD—standard deviation, IQR—interquartile range, INR—international normalized ratio.

**Table 5:** Summary of comparative clinical outcomes between minimally invasive LVAD implantation and full median sternotomy.

Outcome	MICS (Mean ± SD; 95% CI; Range)	FS (Mean ± SD; 95% CI; Range)	Studies Reporting MICS Advantage
30-day mortality	4.80 ± 4.17% (4.12–5.48); 0–13.6	12.06 ± 1.96% (11.87–12.25); 10.3–14.9	Jawad 2021 [27], Özer 2020 [31], Sun 2024 [37], Riebandt 2021 [29], Reichart 2019 [28], Ayers 2020 [35]
6-month survival	89.51 ± 4.39% (88.46–90.56); 84–93	75.48 ± 1.94% (74.98–75.98); 73–77	Gosev 2019 [34], Gosev 2024 [38]
Reoperation (bleeding)	8.03 ± 6.45% (7.29–8.78); 0–23.3	12.60 ± 4.83% (12.09–13.11); 6.8–23.3	Jawad 2021 [27], Gosev 2019 [34], Özer 2020 [31], Antonczyk 2024 [33]
Time in ICU (days)	6.30 ± 2.36 (6.07–6.53); 3.7–13.5	8.39 ± 2.31 (8.18–8.59); 5.4–13.5	Jawad 2021 [27], Ayers 2020 [35], Sun 2024 [37], Reichart 2019 [28], Özer 2020 [31]
Infectious complications	20.27 ± 17.74% (18.35–22.18); 0–44	29.93 ± 14.83% (28.38–31.48); 5.5–41.7	Antonczyk 2024 [33], Kervan 2021 [30], Reichart 2019 [28], Jawad 2021 [27], Gosev 2019 [34]
RVF frequency	17.96 ± 4.89% (17.37–18.55); 7–21.6	22.44 ± 9.40% (21.65–23.23); 10.3–29.9	Saeed 2021 [36], Gosev 2019 [34], Gosev 2024 [38], Jawad 2021 [27], Sun 2024 [37]
Using RVAD	10.03 ± 3.71% (9.56–10.50); 0–13.7	14.29 ± 8.71% (13.55–15.04); 5.4–50	Saeed 2021 [36], Gosev 2019 [34], Gosev 2024 [38], Jawad 2021 [27]

**Table 6:** Specific Features of MICS in High-Risk LVAD Patients.

Authors	Sample Size (MICS/STX)	% With Prior Cardiac Surgery (MICS)	Indication for MICS Approach	Use of Peripheral Cannulation	Cardiopulmonary Bypass (CPB) Duration (min, MICS)	Need to Convert to STX	Comments on Surgical Complexity
Reichart et al. (2018) [28]	22/48	18.2%	Adequate pulmonary function; no concomitant surgery	72.7% femoral access	Not specified	0%	All MICS cases elective; no conversions
Antonczyk et al. (2024) [33]	26/47	54%	Redo patients; high comorbidity	92% femoral artery & 100% vein	59.5 min (median)	0%	Higher OR time; more platelet/FFP transfusions

## Data Availability

The raw data supporting the conclusions of this article will be made available by the authors on request.

## Supplementary Materials

The following supporting information can be downloaded at: https://www.mdpi.com/article/10.3390/medsci13030173/s1. A PRISMA 2020-compliant flow diagram and the completed PRISMA checklist.
